# A Challenging Case of Disseminated Subcutaneous Mycosis from Inner Rio de Janeiro State, Brazil

**DOI:** 10.4269/ajtmh.17-0361

**Published:** 2017-11-08

**Authors:** Walter de Araujo Eyer-Silva, Guilherme Almeida Rosa da Silva, Carlos José Martins

**Affiliations:** Hospital Universitário Gaffrée e Guinle, Centro de Ciências Biológicas e da Saúde, Universidade Federal do Estado do Rio de Janeiro (UNIRIO), Rio de Janeiro, Brazil

A 38-year-old Brazilian man was referred to our hospital because of widespread cutaneous lesions of 10-months duration. Initial lesions emerged on the face and were taken as acne. Multiple lesions followed, and only the scalp was spared. He looked for medical advice when the feet lesions became painful and led to difficulty in walking. The patient lived alone under conditions of extreme poverty in a rural area 200 km far from the city of Rio de Janeiro. Because there was no sewage disposal system, he used to dig cesspits nearby, often using his own hands. He did not have direct contact with cats, but some cats did wander around his dwelling.

**Figure 1.**
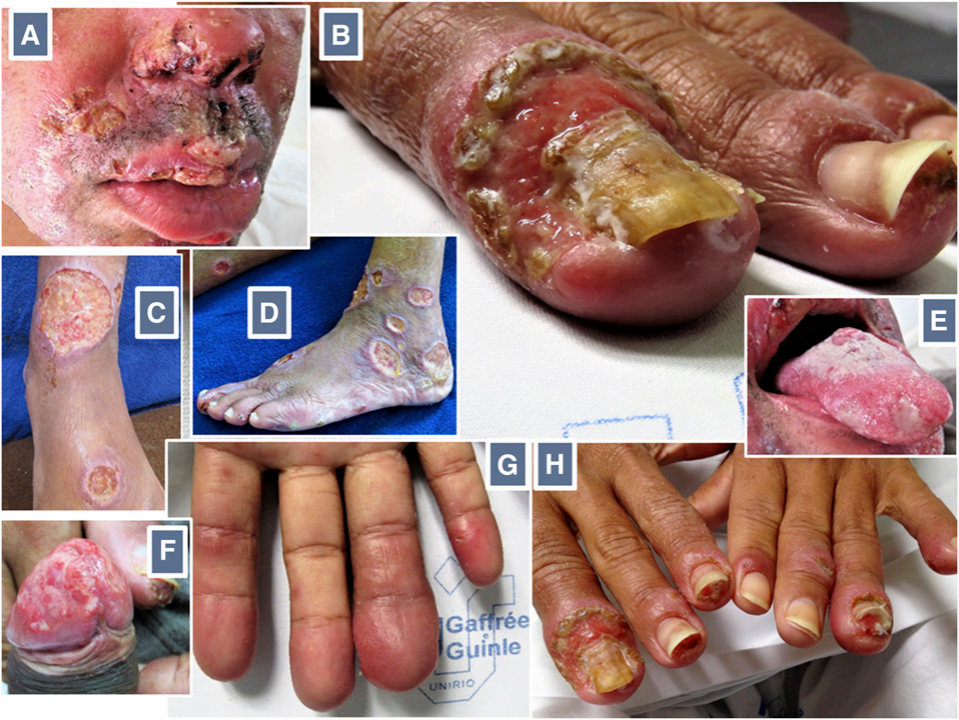
Multiple yellowish or hemorrhagic crust-covered lesions on the face, including the lips and *ala nasi* (**A**). Onychauxis (thickening and transverse ridging) of the right index fingernail, with a large surrounding area of paronychia (**B**). Multiple cutaneous ulcers of varying sizes, some surrounded by violaceous erythema and rolled borders (**C**, **D**). Shallow, fibrin-covered ulcers on the dorsum of the tongue (**E**). Ulcerative balanitis (**F**). Sausage-shaped fingers (**G**). Nail dystrophy and distal detachment from the nail bed (onycholysis) of several fingernails, with a bizarre aspect (**H**). This figure appears in color at www.ajtmh.org.

Clinical examination revealed a wasted, chronically ill patient with multiple tegumentary ulcerated lesions of variable sizes (Figure 1). Larger lesions drained a seropurulent discharge. Among the multiple lesions on the face, those on the lips and *ala nasi* were covered with a yellowish or hemorrhagic crust. There was balanitis, with a large painless ulcerated lesion covering the glans penis, and glossitis, with shallow ulcers on the dorsal aspect of the tongue. The helices and earlobes were reddened and infiltrated. Onychomycosis of multiple nails, paronychia, nail dystrophy, areas of onycholysis, hapalonychia, xantonychia, and onychauxis were recorded. Multiple digits were reddened, enlarged, and sausage-shaped, mainly over the distal phalanges.

**Figure 2.**
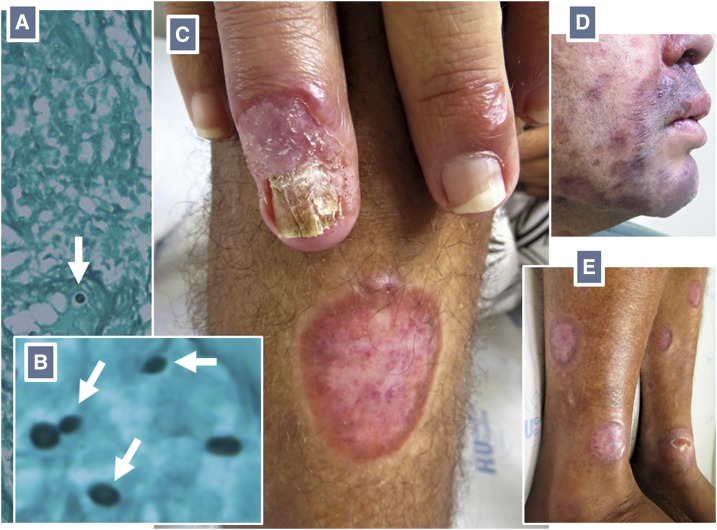
Grocott’s methenamine silver stain unmasks few spherical (**A**) and cigar-shaped yeastlike structures with narrow-based budding (**B**) consistent with *Sporothrix* spp. (white arrows) on a skin biopsy section. Clinical resolution of the lesions after treatment (**C–E**). This figure appears in color at www.ajtmh.org.

Laboratory evaluations were remarkable for a diagnosis of human immunodeficiency virus (HIV) infection, with a CD4 cell count of 249 mm^−3^. He tested negative for syphilis and viral hepatitis. There was no evidence of bone lytic lesions or systemic disease. Histopathological analyses of biopsied tissue revealed an architectural pattern of subcutaneous mycosis, but very few fungal elements were found, a pattern suggestive of sporotrichosis.^1^ These were spherical and cigar-shaped yeastlike structures, which is suggestive of *Sporothrix* spp. (Figure 2). Samples for fungal cultures (collected after initiation of antifungal therapy) were negative. A diagnosis of disseminated subcutaneous mycosis, most probably sporotrichosis, was made.

Sporotrichosis is a neglected opportunistic infection in HIV-infected patients in southeast Brazil.^2,3^ It is transmitted mainly by traumatic inoculation of fungal elements.^4^ A daily regimen of amphotericin B deoxycholate was offered, starting with escalating doses, until a cumulative dose of 1 g was reached. Highly active antiretroviral therapy (HAART) was also started. Paradoxical worsening of the lesions (immune reconstitution inflammatory syndrome) developed 4 weeks later and was treated with corticosteroids.^5^ Three months later he was discharged without active lesions and on oral itraconazole and HAART.
